# Long-Term Live Cell Imaging and Automated 4D Analysis of *Drosophila* Neuroblast Lineages

**DOI:** 10.1371/journal.pone.0079588

**Published:** 2013-11-08

**Authors:** Catarina C. F. Homem, Ilka Reichardt, Christian Berger, Thomas Lendl, Juergen A. Knoblich

**Affiliations:** 1 Institute of Molecular Biotechnology of the Austrian Academy of Sciences, Vienna, Austria; 2 Research Institute of Molecular Pathology, Vienna, Austria; 3 Gregor Mendel Institute, Austrian Academy of Sciences, Vienna, Austria; Institute for Research in Biomedicine, Spain

## Abstract

The developing *Drosophila* brain is a well-studied model system for neurogenesis and stem cell biology. In the *Drosophila* central brain, around 200 neural stem cells called neuroblasts undergo repeated rounds of asymmetric cell division. These divisions typically generate a larger self-renewing neuroblast and a smaller ganglion mother cell that undergoes one terminal division to create two differentiating neurons. Although single mitotic divisions of neuroblasts can easily be imaged in real time, the lack of long term imaging procedures has limited the use of neuroblast live imaging for lineage analysis. Here we describe a method that allows live imaging of cultured *Drosophila* neuroblasts over multiple cell cycles for up to 24 hours. We describe a 4D image analysis protocol that can be used to extract cell cycle times and growth rates from the resulting movies in an automated manner. We use it to perform lineage analysis in type II neuroblasts where clonal analysis has indicated the presence of a transit-amplifying population that potentiates the number of neurons. Indeed, our experiments verify type II lineages and provide quantitative parameters for all cell types in those lineages. As defects in type II neuroblast lineages can result in brain tumor formation, our lineage analysis method will allow more detailed and quantitative analysis of tumorigenesis and asymmetric cell division in the *Drosophila* brain.

## Introduction

The simplicity of the *Drosophila* central nervous system (CNS) and the variety of genetic tools to modify and monitor cell behavior make it an ideal system to study mechanisms of neurogenesis. The *Drosophila* CNS undergoes an embryonic and a post-embryonic period of development. During each period, *Drosophila* neuroblasts (NBs) divide asymmetrically to generate one larger self-renewing and a smaller cell that differentiates into neurons and glia after a limited number of transit amplifying divisions [1]. NBs differentially segregate cell fate determinants to both daughter cells to determine their distinct cell fates. The asymmetric cell division machinery is conserved among all types of NBs and its establishment is independent from extrinsic factors since NBs are capable of dividing asymmetrically in cell culture in the absence of a niche [2-8]. Larval NBs generate an intrinsic axis of polarity by localizing apical and basal polarity proteins on opposite sides of the cell cortex. The Par complex proteins Par3/Bazooka (Baz), Par 6 and atypical protein kinase C (aPKC) localize to the apical side and are inherited by the self-renewing NB [9,10]. The cell fate determinants Numb, Prospero (Pros) and Brain tumor (Brat) localize to the opposite side at the basal cortex and, through binding to mediator proteins such as Miranda (Mira) and Partner-of-Numb (Pon), segregate into the differentiating daughter cell [11,12]. Once inherited by the GMC, Numb, Pros and Brat inhibit self-renewal and promote cell cycle exit and differentiation [13-16].

Based on their lineage, central brain NBs can be subdivided into two types. Around 200 type I NBs divide to self-renew and to generate a GMC that divides once into two neurons or glia [17-20]. In contrast, the 16 type II NBs that are found per brain, generate multiple neurons in a more complex lineage. They are more susceptible to defects in asymmetric cell division. As such defects often cause tumor formation, type II NBs are an attractive model system for studying mechanisms of self-renewal and fate commitment and their connections to tumorigenesis.

Clonal analysis has indicated that the number of progeny generated by type I and type II NBs is similar during the first 24 hours. After 48 hrs, however, type II lineages dramatically increase in cell number while mitotic indices of both type I and type II NBs are equal [21]. From this it has been concluded that type II NBs generate a transit-amplifying population that only becomes mitotically active after a maturation period. This transit amplifying population has been called intermediate neural progenitor (INP) and expresses the type I NB characteristic transcription factors Asense (Ase) and Deadpan (Dpn) after a transient maturation period [22,23]. Additionally, mature INPs also express Earmuff (Erm), a transcription factor promoting Pros-dependent termination of INP proliferation [24]. Mature INPs are capable of self-renewal and can generate a GMC, which then divides to generate two neurons or glia [22]. So far, live cell imaging analysis has not allowed verifying the type II lineage in real time, despite the fact that multiple methods exist for real-time analysis of dividing *Drosophila* NBs [3-5]. Although these approaches have enabled significant insight into the mechanisms of asymmetric cell division and centrosome biology, the short-term nature of these cultures has prevented their use for lineage analysis [7,25-30]. On the other hand neural tissues can be cultured for a long time and remain mitotically active, suggesting that an approach allowing for long-term imaging of dividing NBs may be feasible [31].

Here we describe a method combining long-term live cell imaging of primary NB cultures from larval *Drosophila* central brain with automated 4D image analysis. The method allows individual cells to be followed by high resolution time-lapse video microscopy. We show that our method can be used to verify NB lineages and determine cell cycle times and growth rates in a quantitative manner. Using this methodology we precisely determine division timings and growth rates for all cell types in central brain NB lineages and thereby establish a firm basis for future more precise analysis of mutant phenotypes.

## Material and Methods

### Fly strains and antibodies

*w* ^1118^ was used as wild-type. The following Gal4-driver lines were used: *ase*-Gal4 [32], UAS-*dicer2* ; *wor*-Gal4 *ase*-Gal80 [33], *ase*-Gal4 UAS-*stinger::GFP* (this study), *wor*-Gal4 *ase*-Gal80 ; UAS-*stinger::RFP* (this study). UAS-Baz^S151A.S1085A^::mGFP [34], UAS-*mCherry::Pon-LD*, UAS-*stinger::GFP* [35], UAS-*stinger::RFP* [35], *R9D11-CD8::GFP* [24,36]. Fly crosses for imaging were generally set up at 29°C to increase UAS/Gal4 expression and fluorescence intensity. 

### Antibodies and immunohistochemistry

The following antibodies were used: rabbit anti-Mira (1:100; [16]); guinea pig anti-Deadpan (against full-length MBP fusion protein, serum, 1:1000) rabbit anti-aPKC (1:500; Santa Cruz Biotechnology), mouse anti-PH3 (1:1000; Cell Signaling Technology).

Fixation and stainings of larval brains were performed as previously described [16]. Cultured cells were fixed in 4% paraformaldehyde (PFA) for 10 min, blocked for 1 hr in PBS with 10% NGS, incubated with primary antibody for 1hr at room temperature, washed 3 times 10 min with PBS, incubated with secondary antibodies for 1hr at room temperature, mounted in anti-fade (SlowFade Antifade Kit-Invitrogen) and imaged immediately. Immunofluorescent images were acquired on either LSM510 or 780 microscopes (Carl Zeiss GmbH).

### Cell Dissociation and Primary cell cultures

Third instar larval were collected and washed once in phosphate-buffered saline (PBS), dissected in supplemented Schneider’s medium (10% fetal bovine serum, 2% Pen/Strep, Schneider’s medium (GIBCO)) and larval brains were collected and washed in cold Rinaldini solution [4]. For cell dissociation, collected brains were incubated in Rinaldini solution with 1 mg/ml collagenase I and 1mg/ml of papain (Sigma Aldrich) for 1 hr at 30°C. Brains were washed twice with Rinaldini solution and once with supplemented Schneider’s medium. Brains were manually disrupted with a pipette tip in 200 μl supplemented Schneider’s medium. The dissociated brains were plated in 0.01% poly-L-lysin-hydrobromide coated glass bottom cell culture dishes (Matek and Invitro Scientific) and allowed to settle for 1 hr at RT. Before imaging, 3 ml of primary cell culture Schneider’s medium (10% fetal bovine serum, 2% Pen/Strep, L-Glutamine 20 mM, L-Glutathione 5μg/ml, Insulin 20 μg/ml, Ecdysone 5μg/ml, Schneider’s medium) was added to the cells and imaging was performed immediately.

### Live imaging

Live imaging of cultured cells was performed using an Ultra View Vox spinning-disc confocal system (Perkin Elmer) installed on an Axio Observer Z1 microscope (Carl Zeiss GmbH). Images were recorded with an Hamamatsu EMCCD 9100-13 camera (Hamamatsu) in 8Bit mode, using 40x/1.3 EC plan-neofluar lens (Zeiss) and 1.2x additional magnification lens mounted in front of the camera. Acquisition of video sequences was done with the Volocity 3D Image Acquisition and Analysis Software (Perkin Elmer); multiple positions were acquired simultaneously. At each position Z-stacks with 1 µm intervals were captured every 3 min. Laser intensity (2 %) and exposure time (10 msec) were adjusted to avoid cytotoxicity. Collected images were deconvolved using Huygens deconvolution suite (SVI). Maximum intensity projections of the deconvolved stacks were compiled and converted to AVI movies with Imaris (Bitplane).

### Automated 4D image analysis

Nuclear sizes were measured by using Definiens, an object based image analysis software (Definiens®). First, a region of interest was defined and subsequently ‘automatic threshold’ was used to identify approximate cell borders. To define nuclei, a Gauss filter was applied and ‘automatic threshold’ was applied a second time. Nuclei that directly contacted each other were separated by defining seed points in the center of each nucleus and by expanding those to the size of the object. To avoid segmenting single nuclei into multiple objects, quality criteria for shape and size were automatically defined for each object. Oversegmented objects were adjusted manually. Borders between detected nuclei were redefined by shrinking and growing, taking intensity values into account. After nuclei segmentation, cell types were defined and linked over time. Voxel numbers of each object were counted and converted into absolute volume size. More detailed explanation on the methodology is available on request.

### Statistical analysis of division timing, cell sizes and cell growth rates

To compare type I and type II NB sizes, the nuclear volumes during the 9 min preceding mitosis were averaged as nuclei reached their maximal size during this time. Nuclear volumes of INPs were determined as the average volume during the 39-45 min after the INP could first be detected. Nuclear volumes of GMCs were determined as the average volume during the 9-15 min after the GMC could first be detected. Growth rates were defined as the inverse ratio between average volumes over 9min after mitosis and 9 min before the next mitosis. Cell cycle lengths in NBs/INPs/GMCs were determined as the time between two subsequent nuclear envelope breakdowns.

## Results and Discussion

### NBs and INPs divide asymmetrically in culture

**Figure 1:**
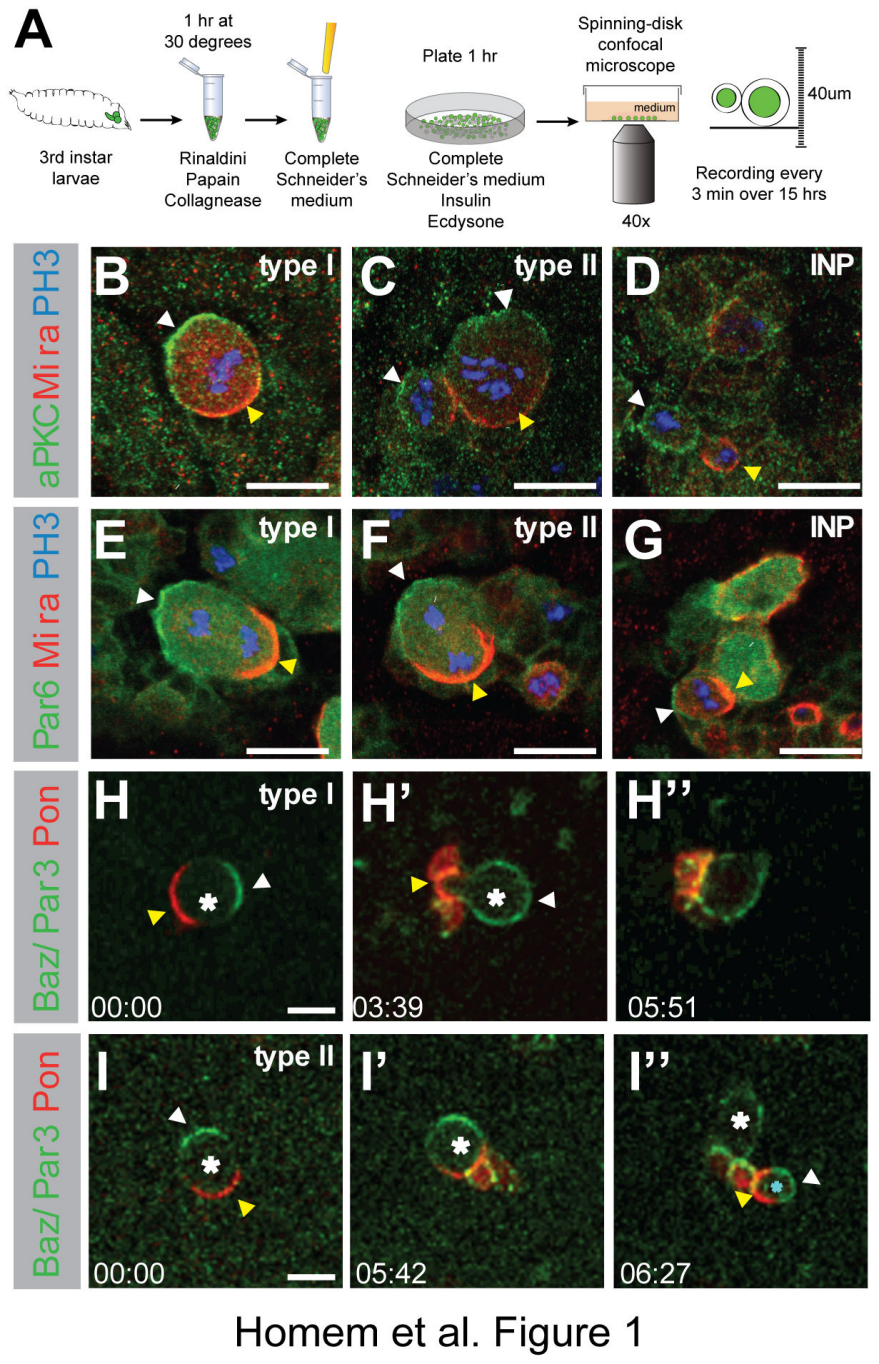
Neuroblasts and INPs divide asymmetrically in culture. (**A**) Schematic of the experimental set-up. (**B**-**G**) *In* *vivo* staining of wild-type 3rd instar larval brains. Type I (B, E), type II NBs (C, F) and INPs (D, G) stained for Mira (red), PH3 (blue) and aPKC (B-D, green). Par 6-GFP (E-G, green) in par 6 mutant background. (**H**, **I**) Single frames from cultured NB time-lapse movies. NBs expressing UAS-Baz^S151A.S1085A^::GFP, UAS-mCherry::Pon-LD. White asterisk labels NB, blue asterisk marks INP. (H) Single frames from movie of type I NB undergoing multiple rounds of division in culture. (I) Single frames from movie of type II NBs undergoing multiple rounds of division in culture. Times in hr:min. (A-H) White arrowheads label apical, yellow arrowheads basal polarity domains. Scale bars, 10 µm.

To monitor type I and type II NB lineages in primary cell culture we isolated NBs by enzymatic digestion and gentle mechanical disruption and plated them on poly-L-lysine coated glass bottom dishes (see Material and Methods section, Figure 1A). *In vivo*, NBs and INPs establish an internal polarity axis, localizing self-renewing Par complex proteins aPKC, Par 6 and Baz to the apical domain (Figure 1B-G) and differentiating factors such as Mira to the basal domain to be inherited by the INP or GMC, respectively (Figure 1B-G) [9,10]. To test whether NBs correctly localized and segregated cell fate determinants in long-term cultures, we followed type I and type II NBs expressing the apical marker Baz-GFP and basal marker mCherry-Pon. To avoid any effects of Baz overexpression we used the previously described non-phosphorylatable Baz^S151A.S1085A^-GFP [34]. Both type I and type II NBs in culture formed an axis of polarity and asymmetrically localized Baz^S151A.S1085A^-GFP to their apical cortex and mCherry-Pon to opposite basal side (Figure 1H, I). Cultured INPs were also capable of asymmetrically distributing apical Baz and basal Pon (Figure 1I, 06:27). In both type I and type II NBs and also in INPs the apical and basal cortical domains were inherited by the proliferating and differentiating cell, respectively (Figure 1H, 03:39, 1I, 05:42, Figure S1A, 06:24 and 09:45, Movie S1). Both type I and type II NBs went through successive rounds of division generating extended lineages (Figure 1H, 05:51, 1I, 06:27). Thus, as *in vivo*, cultured type I, type II NBs and INPs asymmetrically distributed apical and basal polarity proteins to result in asymmetric progeny cell fates.

### NBs Generate Correct Lineages in Culture

**Figure 2:**
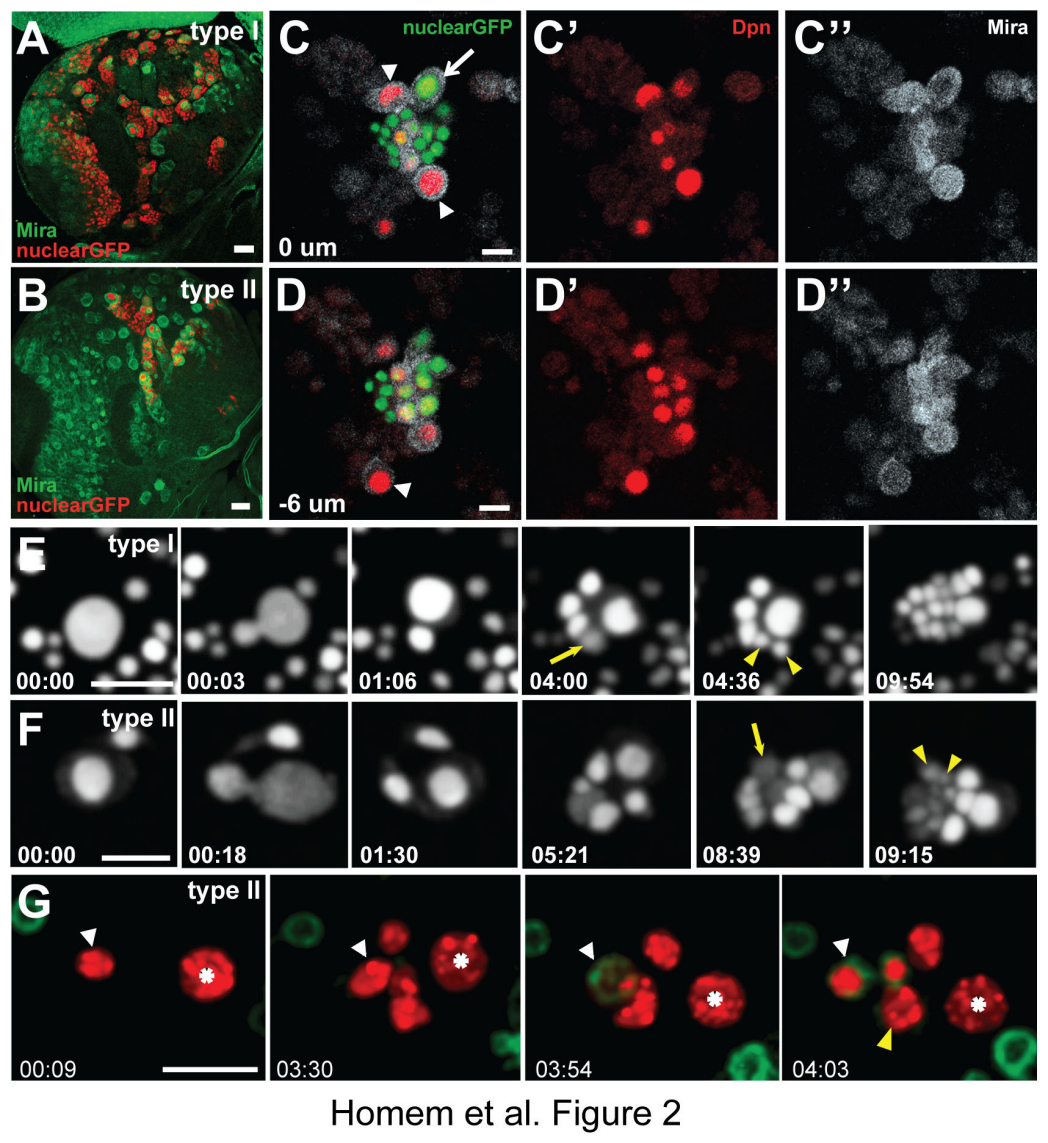
Neuroblasts generate *in vivo*-like lineages in culture. (**A**, **B**) Drosophila larval brain lobe expressing type I (A) or type II (B) specific nuclear GFP (red) stained for Mira (green). Scale bars, 20 µm. (**C**, **D**) 20-hr NB primary cell culture stained for Dpn (red) and Mira (white). Type II NBs are identified by nuclear GFP (green, arrow), whereas type I NBs are GFP negative (arrowheads). Cell culture stainings are represented in two layers. (**E**, **F**) Image time course from representative movies capturing a dividing type I (E) or type II (F) NB. Arrows mark GMC (E) or INP (F) shortly before division. Arrowheads label daughter cells shortly after the GMC (E) or INP (F) divided. Note that the GMC leads to two daughter cells equal in size, whereas the INP divides asymmetrically generating two daughter cells that differ in size. (**G**) The type II NB (asterisk) generates INPs that express *R9D11-CD8::GFP* shortly before they divide (1st INP white arrowhead, 2nd INP yellow arrowhead). Times are in hr:min. Scale bars, 10 µm.

In order to test whether NBs were able to generate correct lineages in cell culture we generated 20-hr cell cultures and analyzed their composition by immunostaining for specific proliferation or differentiation markers (Figure 2C, D). To identify the correct types of NBs, we used nuclear GFP (UAS-*stinger::GFP*; [35]) expressed under the control of the *Ase* promoter (*ase*-Gal4; type I specific, Figure 2A) or under the control of the *Worniu* promoter simultaneously inhibited in cells expressing *Ase* (*wor*-Gal4 *ase*-Gal80; type II specific, Figure 2B). NBs of both type I and type II were positive for the proliferation markers Dpn (Figure 2D’,E’) and Mira (Figure 2D’’,E’’). Type II NBs also displayed Dpn and Mira positive progeny, indicating that some daughter cells exhibited self-renewing NB-like identity. In contrast, all type I NB progeny were Dpn and Mira negative. From these results we can conclude that NBs in primary cell culture form correct lineages. 

To analyze NB lineage progression over time we used live cell imaging microscopy. NBs were isolated and immediately imaged by acquiring 40 µm thick stacks every 3 min over a time period of 24 hrs in a spinning-disk confocal microscope (Figure 1A). Both type I and type II NBs divided asymmetrically and generated daughter cells that differed in size (type I Figure 2E, 00:03, Movie S2; type II Figure 2F, 00:18, Movie S3). In both lineages, the bigger cell resembling the self-renewing NB divided multiple times, whereas the smaller cell remained quiescent first before entering mitosis. The smaller type I NB daughter cell divided symmetrically and generated two daughter cells equal in size (Figure 2E 04:36). In contrast, the daughter cell of a type II NB divided asymmetrically and generated two daughter cells that differed in size (Figure 2F, 09:15). These observations are consistent with the NB behavior *in vivo* at which GMCs of type I NBs divide symmetrically and generate neurons, whereas INPs of type II NBs divide asymmetrically to self-renew and simultaneously generate a more differentiated GMC [21]. Once born, INPs undergo a maturation period before entering mitosis. During this maturation period INPs activate the expression of the transcription factor Erm [24]. To investigate if INPs undergo the same maturation period *in vitro* as they do *in vivo* we imaged type II NB lineages expressing membrane tethered GFP under the control of the *Erm* promoter (*R9D11-CD8::GFP*, [36]). The type II NB, identified by type II specific expression of nuclear RFP, lacked *Erm* driven expression of *CD8::GFP* (Figure 2G, Movie S4). In contrast, the small daughter cell of the type II NB initiated *CD8::GFP* expression during its maturation shortly before entering mitosis (Figure 1H). From our analysis, we conclude that type II NBs during live cell imaging of primary cell culture generate correct *in vivo*-like progeny.

### Automated 4D image analysis and visualization

**Figure 3:**
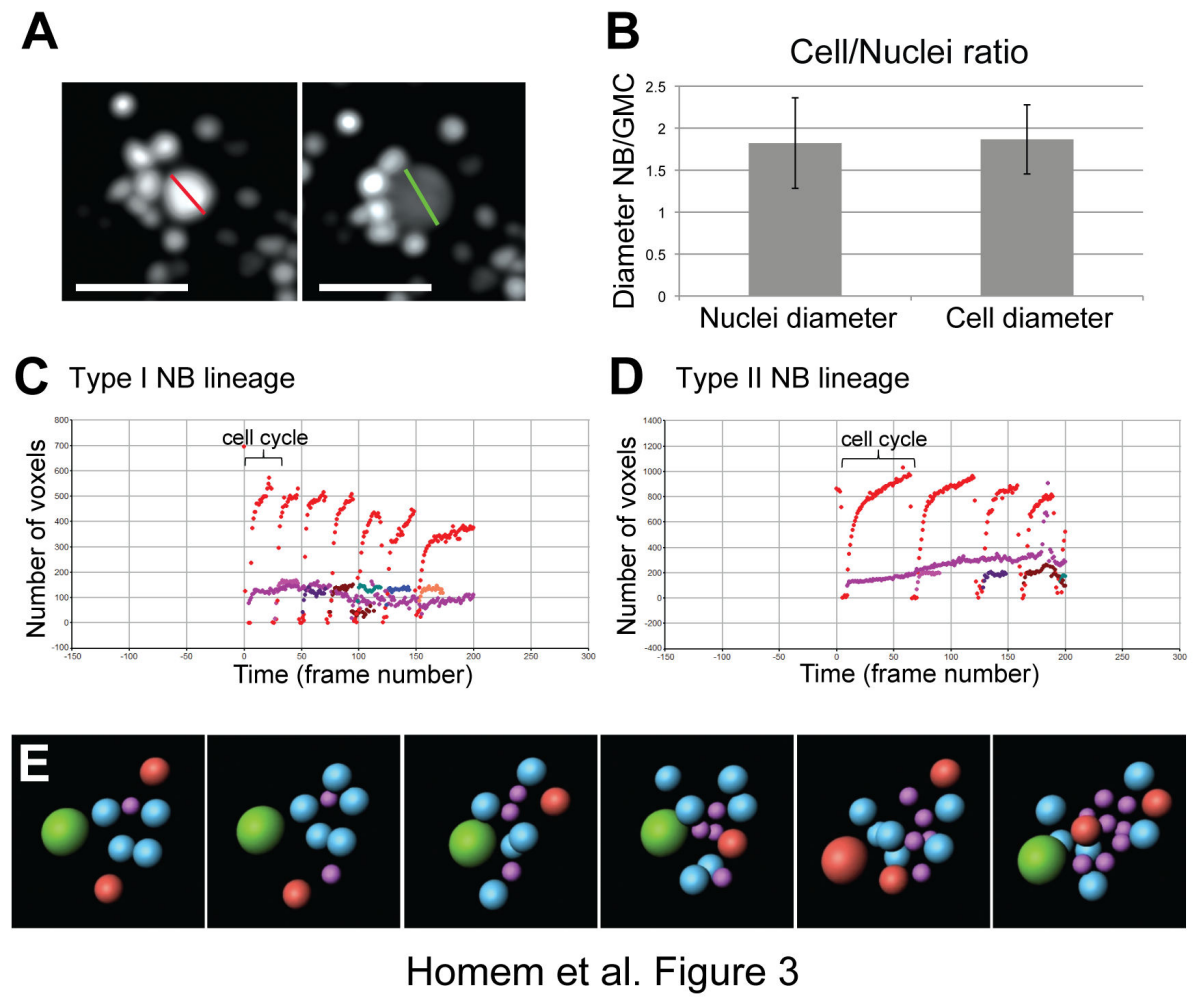
Automated 4D image analysis. (**A**) Type I lineages expressing nuclear GFP. Nuclei diameters were measured before cell division (red line). Cell diameters were obtained by measuring cell diameter when nuclear GFP labeled the entire cell right after nuclear breakdown (green line). Scale bar, 10 µm. (**B**) Size ratio of type I NB/GMC estimated with nuclei diameter *vs.* size ratio estimate with cell diameter (n=5). Error bars represent standard deviation. (**C**, **D**) Examples of output images of type I (C) and type II (D) NB lineage analysis by Definiens. One frame equals three minutes. Red marks the NB, purple marks the GMC (C) or INP (D); the remaining colors mark neurons (C) or GMCs (D). Note that only the first INP was followed throughout entire INP cell cycle, whereas the following born INPs were followed only for a few frames. (**E**) Representative stills from type II NB lineage live imaging movie modified by IMARIS. Green ball represents the type II NB, blue balls represent INPs, purple balls represent GMCs. Red balls are in mitosis.

To accurately follow the dynamics of neural lineages, we developed an automated 4D image software. The methodology is based upon the Definiens Software Suite, an object based image analysis software (Definiens®) Both membrane tethered and cytosolic GFP did not allow continuous assignment of individual cells through many mitotic divisions. To overcome this limitation, we used nuclear GFP, which could be accurately detected and tracked by our image software. We first determined whether nuclear size provides a correct estimate for cell size in NB lineages by calculating the ratio between cell/nuclear diameters in NBs and their daughter cells. Nuclear diameters were measured just before cell division and whole cell diameters were measured after nuclear breakdown, when GFP was dispersed throughout the entire cell (Figure 3A). The ratios between NB/GMC nuclei diameter and NB/GMC cell diameter were constant (Figure 3B). Thus, we used nuclear sizes to determine cell growth rates and to compare sizes of the different cell types within a neural lineage. 

Live cell imaging was performed using a spinning-disk confocal microscope and movies were recorded using the Volocity Software. Lineages were recorded over a time period of 24 hrs and NBs and daughter cells were dividing appropriately (Movie S5). As the NB cell cycle length became increasingly variable over time, only the first 200 frames (10 hrs) were used for further analysis.

Image stacks were deconvolved using the Huygens deconvolution suite (SVI) and subsequently imported to Definiens® software. Using various segmentation tools of Definiens software nuclei were defined and tracked over time (see Material and Methods). The software could accurately follow and measure nuclear volumes of all cells in NB lineages. The resulting voxel numbers for cell nuclei of both type I and type II lineages were plotted over a time period of 200 frames (3min/frame; Figure 3C, D). NBs grew until they reach their maximum size followed by division. Each cell cycle could be identified by the gaps between each growth period. These gaps were due to the nuclear envelope breakdown, which led to a uniform distribution of nuclear GFP throughout the cell causing a strong decrease in signal intensity, which was no longer recognized by the software (Figure 3C, D). To visualize three-dimensional neural lineage formation, deconvolved image stacks were imported to IMARIS software and nuclei surfaces were defined over time using IMARIS tools. Different cell types were labeled by different colors and cells in mitosis were highlighted (Figure 3E, Movie S6).

### Cell cycle timing and cell growth rates

**Figure 4:**
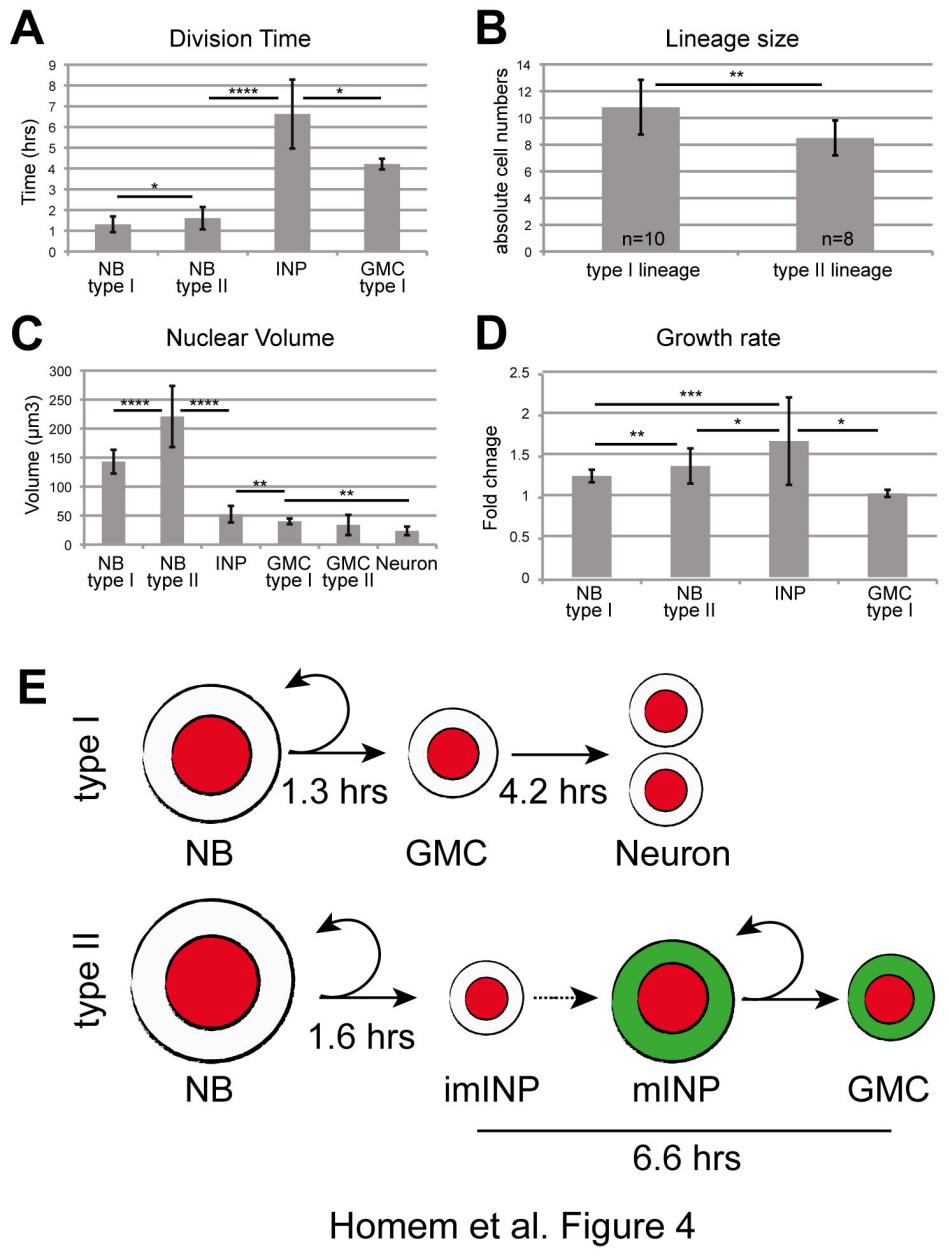
Quantitative analysis of type I and type II neural lineages. (**A**) Frequency of divisions of type I and type II NBs, INPs and GMCs (n=29, n=31, n=6, n=6 respectively). Time measured in hours between consecutive nuclear breakdowns. (**B**) Absolute cell numbers type I and type II lineages counted after 10-hr recording period. (**C**) Nuclei volumes from all cell types of type I and type II lineages. NB type I n=30, NB type II n=31, INP n=34, GMC type I n=5, GMC type II n=6, neuron n=5. (**D**) Growth rates from birth to division of type I and type NBs, INPs and GMCs. NB type I n=30, NB type II n=34, INP n=6, GMC type I n=4) (A-D) Bars represent standard deviation. Statistical analysis done using T-test. *p<0.05; **p<0.01; ***p<0.001; ****p<0.0001. (**E**) Graphic representation of type I and type II lineages. Diameter ratios between the lineages and the different cell types is according to experimental measurements. GMC, ganglion mother cell; imINP, immature intermediate neural progenitor; mINP, mature INP; NB, NB. Red color represents nuclei, green color represents Earmuff expression.

Clonal analyses of NB lineages can give information about NB cell cycle and lineage expansion over time; however, mitotic indices of NB progeny are difficult to determine due to the increasing lineage complexity. Thus, precise cell cycle analysis of the different cell types beside the NB within a lineage requires live cell imaging analysis. From the automated image analysis we observed type I NBs dividing every 1.3 hrs, while type II NBs dividing every 1.6 hrs (Figure 4A). The timing is consistent with previous clonal *in vivo* analysis (1.5 hrs for both NB types, [21]) and live imaging of type II NB clones in whole brain explants [37]. Clonal analysis predicted that INPs would require a maturation period of 3 - 9 hrs before starting to divide. Clonal live cell imaging, on the other hand, suggested that INPs divide every 2 hrs [21,37]. This difference can be explained because every new born INP needs to undergo maturation before its first division. Once INP maturation is complete, the cell cycle is shorter. Bello et al. analyzed dividing INPs far away from the NB. Most likely, these were already in their second cell cycle and maturation was completed. To characterize division timings of INPs we analyzed cell cycle timing of newly born and also mature INPs. We found that newly born INPs underwent their first division 6.6 hrs after birth (Figure 4A), whereas mature INPs divided every 2 - 3 hrs (Figure S1, Movie S1, Movie S5). Thus, our findings are consistent with previous observations [21,37].

GMCs of type I NBs divided 4.2 hrs post-generation (Figure 4A). Short-term (24 hrs) clonal analysis has shown that type I and type II NBs first generate approximately the same number of progeny (type I 28 cells *vs.* type II 26 cells), whereas long-term (48 hrs) type II NB clones contain considerable more cells than type I (type I 58 cells *vs.* type II 131 cells) [21]. Consistently, during a recording period of 10 hrs we found type I lineages containing 11 cells and type II lineages 9 cells (Figure 4B). Thus, we conclude that neural lineage timing in cell culture perfectly resemble what has been shown by clonal *in vivo* analysis and in addition, times could be determined more accurately.

The difficulty of performing precise cell volume analysis in fixed brain tissue, due to the amorphous shape of cells within a lineage, has so far only allowed obtaining approximate cell sizes [21,37]. Taking advantage of the optimized long-term NB culture and software analysis method, we have followed cell sizes of both type I and type II NBs and their progeny. The automated analysis revealed that the type II NB was 1.5-fold bigger than the type I NB (Figure 4C). The small daughter cell of the type I NB was 3.6- the one of the type II NB 4.2-fold smaller compared to its respective parental NB (Figure 4C). The self-renewing INP was 1.5-fold larger than its more differentiated progeny, the type II GMC. The size of a type I or type II GMC was not significantly different and neurons were the smallest cell type in both lineage types (Figure 4C). Larval NBs regrow to their original size after each asymmetric division, and it has been hypothesized that the NB regrowth capacity is correlated to self-renewal capacity [38]. During the INP maturation period, several transcriptional changes occur. By the end of this period INPs are *Dpn* ^*-*^ *Ase* ^*+*^ *Erm* ^*+*^ and capable of dividing and self-renewing [22,24]. It is however unknown if and to what extend INPs regrow during the maturation period. To analyze this we determined growth rates of NBs, INPs and GMCs. Automated image analysis revealed that type I and type II NBs grow 1.2- and 1.4-fold, respectively (Figure 4D). The difference in growth rate between the two types of NB can be explained by the different sizes of the daughter cells. The type II NB generates INPs that are 1.5-fold bigger than the GMCs generated by type I NBs. Thus, to maintain their original size, type II NBs have to grow more than type I NBs. GMCs of type I NBs do not grow (Figure 4D). Surprisingly, the growth rate of INPs was even larger compared to NBs as they grow on average by 1.7-fold (Figure 4D). Although INPs grow more, they do this at a slower rate (0.1 μm^3^/min) than type I NB (0.69 μm^3^/min) or type II NBs (0.92 μm^3^/min).

## Conclusions

The majority of neurons in the *Drosophila* adult brain are generated during the larval period in a second wave of neurogenesis. Neural lineages have been studied extensively, but detailed knowledge about their behavior and progression is still sparse. *In vivo* studies have to deal with great structural complexity and do not always allow distinguishing between cell autonomous and non-cell autonomous processes. To complement *in vivo* neurogenesis studies, we have developed a long-term primary cell culture system combined with an automated 4D image analysis, which allowed for studying neural lineages in great detail. We generated primary cell cultures of *Drosophila* larval brains and showed that both type I and type II NB lineages were formed accurately in culture. Long-term culture and image analysis allowed for determining cell cycle timing and cell growth rates of NBs and their progeny. Due to their transit-amplifying progeny, which makes them more susceptible to tumorigenesis, type II NB lineages provide a great model to study mechanisms in stem cell biology. We exploited the long-term live cell imaging method introduced here to analyze type II NBs and INPs in great detail. Like *in vivo*, cultured INPs undergo a set of maturation steps, and asymmetrically divide to self-renew and generate more differentiated daughter cells. Thus, our long-term cell culture system perfectly resembles *in vivo* neural lineage behavior. Now, this system facilitates experiments to analyze larval brain neurogenesis, e.g. proliferation control and the contribution of extrinsic and intrinsic factors, and provides a convenient tool to study mutants and genetically manipulated neural lineages. Previous studies have demonstrated that centrosomes are asymmetrically inherited by the NB and its daughter cells [5,39]. The method that we described here will allow the analysis of long-term centrosome inheritance, for example through the entire lineage and in INPs. The system we have established can also be applied for studies of polarity establishment in stem cells and their progeny.

## Supporting Information

Figure S1 **Single frames from movie of type II NBs undergoing multiple rounds of division in culture.** NBs expressing UAS-Baz^S151A.S1085A^::GFP, UAS-mCherry::Pon-LD. Asterisk marks the same INP dividing twice. Arrowheads label the daughter cells of the INP. Note, that the daughter cell inheriting the apical domain (Baz/Par3) divides again. Times in hr:min. Scale bar, 10 µm.
(TIF)

Movie S1 **Type II NB lineage in cell culture expressing UAS-Baz^S151A.S1085A^::GFP and UAS-mCherry::Pon-LD.** The INP inheriting the apical domain (Baz/Par3, 06:24) divides again at 09:25.
(AVI)

Movie S2 **Type I NB lineage in cell culture expressing nuclear GFP by *ase-*Gal4.**
(AVI)

Movie S3 **Type II NB lineage in cell culture expressing nuclear GFP by *wor*-Gal4 *ase*-Gal80.**
(AVI)

Movie S4 **Type II NB lineage in cell culture expressing nuclear RFP by *wor*-Gal4 *ase*-Gal80 and membrane tethered GFP by *erm*-Gal4.**
(WMV)

Movie S5 **Type II NB lineage in cell culture expressing nuclear GFP by *wor*-Gal4 *ase*-Gal80 recorded over a time period of 23 hrs.**
(AVI)

Movie S6 **IMARIS modulated movie illustrating the formation of a type II NB lineage.** The green ball represents the NB, blue balls represent INPs and purple balls represent GMCs. Cells undergoing division are highlighted in red.
(MP4)
