# Supplementary figures and images for: Long-Term Live Cell Imaging and Automated 4D Analysis of Drosophila Neuroblast Lineages
Source: PLoS One. 2013 Nov 8;8(11):e79588. doi: 10.1371/journal.pone.0079588 (PMC3832664; doi:10.1371/journal.pone.0079588)

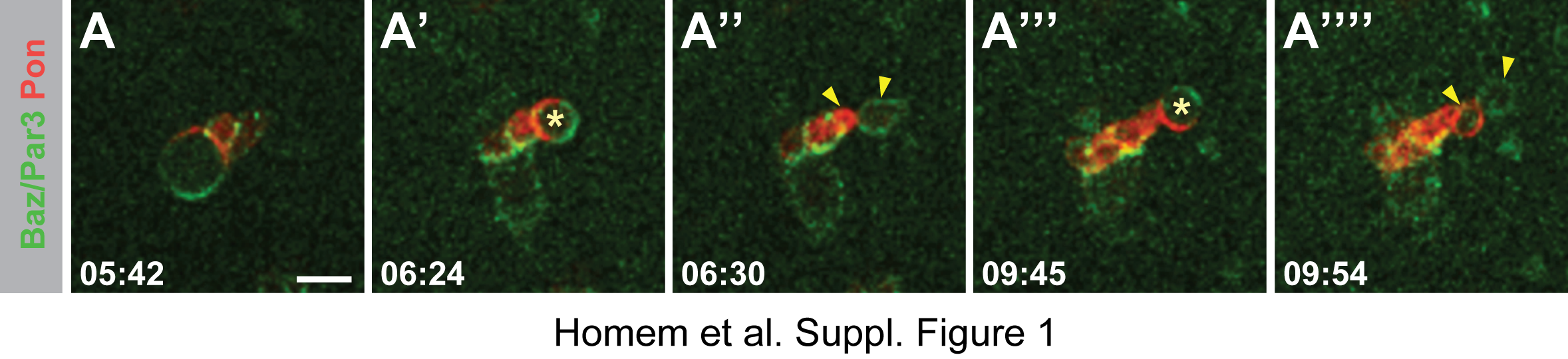

Supplement: Figure S1 — Single frames from movie of type II NBs undergoing multiple rounds of division in culture. NBs expressing UAS-BazS151A.S1085A::GFP, UAS-mCherry::Pon-LD. Asterisk marks the same INP dividing twice. Arrowheads label the daughter cells of the INP. Note, that the daughter cell inheriting the apical domain (Baz/Par3) divides again. Times in hr:min. Scale bar, 10 µm. (TIF) [file pone.0079588.s001.tif]
